# Validating and Comparing C-TIRADS, K-TIRADS and ACR-TIRADS in Stratifying the Malignancy Risk of Thyroid Nodules

**DOI:** 10.3389/fendo.2022.899575

**Published:** 2022-06-17

**Authors:** Qingfang Chen, Mingnan Lin, Size Wu

**Affiliations:** Department of Ultrasound, The First Affiliated Hospital of Hainan Medical University, Haikou, China

**Keywords:** thyroid nodule, malignancy, ultrasound, thyroid imaging reporting and data system (TIRADS), C-TIRADS, K-TIRADS, ACR-TIRADS

## Abstract

The thyroid imaging reporting and data system (TIRADS) was proposed by experts for optimal ultrasound evaluation of malignancy risk of thyroid focal lesions. There are several versions of TIRADS, some of them have been validated sufficiently, and the others have not been well assessed. In this study, a recently launched Chinese version of TIRADS (C-TIRADS) for malignancy risk stratification of thyroid nodules was validated, and the performance was compared to that of the Korean TIRADS (K-TIRADS) and American College of Radiology(ACR) TIRADS (ACR-TIRADS). Archives of 2177 patients who had undergone thyroid ultrasound examination, coarse needle tissue biopsy and/or surgery were reviewed, and 1978 patients with 1982 thyroid nodules were assessed according to the three TIRADSs. The histopathology was taken as the golden standard. The results showed the 1982 thyroid nodules were consisted of 1306 benign nodules and 676 malignant nodules. The malignancy risk accounted for 1.09%, 2.14%, 10.34%, 49.28%, 88.19% and 85.29% of the total nodules that were categorised as C-TIRADS 2, 3, 4A, 4B, 4C and 5, respectively; 0.00%, 1.64%, 2.87%,18.71% and 82.22% of the total nodules that were categorised as ACR-TIRADS 1, 2, 3, 4 and 5, respectively; 0.85%, 3.27%, 24.27% and 80.96% of the total nodules that were categorised as K-TIRADS 2, 3, 4 and 5, respectively. The correlation between the category of TIRADS and percentile of malignancy was 0.94 in the C-TIRADS, 1.00 in the ACR-TIRADS, and 1.00 in the K-TIRADS, respectively. The highest values of accuracy(AUC) *of* ROC curves *of* C-TIRADS 4B, K-TIRADS 5 and ACR-TIRADS 5 were taken as the cut-off values for risk stratification, respectively. The sensitivity, specificity, positive and negative predictive values and AUC by C-TIRADS 4B, K-TIRADS 5 and ACR-TIRADS 5 for malignancy risk stratification of thyroid nodules were 90.83%, 84.23%, 74.88% and 94.66% and 0.88, respectively; 83.58%, 89.82%, 80.95%, 91.36% and 0.87, respectively; and 85.50%, 90.35%, 82.10%, 92.33% and 0.88, respectively (*P*>0.05 for all). We concluded that the C-TIRADS has excellent performance in the malignancy risk stratification of thyroid nodules by the optimized cut-off value, which is comparable to that in K-TIRADS and ACR-TIRADS.

## Introduction

Thyroid nodules are common in adults, and they have a prevalence of 19%–68%, depending on the population investigated (1–5). This includes a malignancy rate between 6.7% and 15% (1, 4, 5). Thyroid nodules may be benign, borderline or malignant lesions, and the prevalence of benign nodules are much more common than malignant nodules (1). Timely detection and accurate diagnosis of thyroid nodules are significant for the clinical management of the patients. However, many patients with malignant nodules have no unique clinical manifestations or laboratory abnormalities before distant metastasis. This makes it difficult to differentiate malignant nodules from benign nodules (1, 6–9).

Color Doppler ultrasound (US) is the imaging modality most frequently used to evaluate thyroid nodules. It has an excellent performance in detecting nodules, but its ability to serve as a basis for stratifying the malignancy risk must be improved (6–9). To improve the efficiency of US diagnosis, Horvath et al. established the first thyroid imaging reporting and data system (TIRADS) based on the US features of thyroid nodules in 2009, and as of 2020, there are eight versions of TIRADS in the world (9). The reasons for multiple TIRADSs are that each one has some advantages. However, they also have limitations, such as the similarities and discordance in terminology and standards in describing and defining the US features of thyroid nodules when different researchers establish their classification systems. The TIRADSs have different aims, so no one system has been widely acknowledged and used, and the effort to validate them remains hot (6–16). The latest version of TIRADS is the Chinese version of TIRADS (C-TIRADS), released in 2020 by The Superficial Organ and Vascular Ultrasound Group of the Society of Ultrasound in Medicine of Chinese Medical Association (9).

There are different categorisation schemes for the different versions of TIRADS. Taking as an example, the ACR-TIRADS recommended by the American College of Radiology(ACR), scoring involves 23 US features with different weightings (8). The categorising schemes of the Korean TIRADS (K-TIRADS) recommended by The Korean Society of Thyroid Radiology and Korean Society of Radiology and C-TIRADS involve fewer US features than that of the ACR-TIRADS, and C-TIRADS uses six dominant US features that are highly suggestive of malignancy or benignity (7, 9). Because the scoring scheme of ACR-TIRADS is based on many US features, it has the strength of being systematic and comprehensive, but it is not easy to apply. If a simpler scheme can be found to be effective, that would be an improvement. Whether a simpler scheme of the C-TIRADS compromises or increases its ability to stratify malignancy risk has not been investigated. The aim of this study was to validate the C-TIRADS and compare the performance of the three TIRADSs for malignancy risk stratification of thyroid nodules.

## Subjects and Methods

### Study Population

A total of 2177 patients who had undergone US thyroid examination, coarse needle biopsy and/or surgery in the First Affiliated Hospital of Hainan Medical University between January 2015 and December 2021 were selected as potential research subjects, and their data were retrospectively reviewed. The inclusion criteria were patients with eligible qualified US images of their thyroid nodule(s) and with histopathological results. The exclusion criteria were US images of thyroid nodules that were of inferior quality or had image numbers that could not display the nodular features fully, or the histopathology of the thyroid nodule was undetermined. A coalescence of nodules was counted as one nodule. If a patient had more than thyroid nodules, the nodule with features suggestive of malignancy or a representative nodule of benign nodules was enrolled. (Four patients had malignant nodules in both lobes of the thyroid.) Finally, 1978 patients with 1982 thyroid nodules were included, and 199 patients with 202 thyroid nodules were excluded.

### Key Points of Assessment by C-TIRADS

According to the C-TIRADS, the assessment of a thyroid and thyroid nodule involves features of nodular composition (architecture), echogenicity, shape (orientation), margin, and echogenic foci (9). The scoring scheme is based on evaluating sonographic features on the basis of different points. One point is added for features that suggest the malignancy risk, and one point is subtracted for features that suggest benignity. The details are as follows. One point is added for each feature of solid composition, that is markedly hypoechoic; taller than wide (A/T≥1); any one or more of the features of ill-defined, irregular, lobulated, and extrathyroidal extension margin; and punctate hyperechogenicity (suspicious calcification). One point is subtracted for feature of hyperechoic foci with a comet-tail artifact. If there are more than one hyperechoic patterns in a nodule, only the feature with highest value is included. The C-TIRADS has eight categories established according to the total value of the thyroid or thyroid nodule. The categories range from a nodule-free thyroid to a benign nodule, a nodule of highly suspicious malignancy, and a malignant nodule confirmed by histology. A nodule-free thyroid is categorised as TIRADS 1; a nodule with a value of -1 is categorised as TIRADS 2; a nodule with a value of 0 is categorised as TIRADS 3; a nodule with a value of 1 is categorised as TIRADS 4A; a nodule with a value of 2 is categorised as TIRADS 4B; a nodule with a value of 3 or 4 is categorised as TIRADS 4C; a nodule with a value of 5 is categorised as TIRADS 5; a nodule with a malignancy confirmed by histopathology is categorised as TIRADS 6; and a simple cystic nodule or a spongy nodule is categorised as TIRADS 2. Sonographic features for scoring are as follows: solid composition = +1, markedly hypoechoic = +1, taller than wide (A/T≥1) = +1, any one or more of the features of ill-defined, irregular, lobulated, and extrathyroidal extension margin = +1, hyperechoic foci with a comet-tail artifact = -1, and punctate hyperechoic foci (suspicious calcification) = +1. If there are more than one hyperechoic patterns in a nodule, only the highest scored is included.

### Key Points of Thyroid Nodular Assessment by the K-TI-RADS (2016)

The K-TIRADS rates the malignancy risk with reference to three suspicious US features: microcalcification, a shape that is taller than wide and a spiculated or microlobulated margin, and other US features of nodular composition and echogenicity (7). K-TIRADS 5 indicates high suspicion of malignancy when there is solid hypoechoic composition with any of the three US features. K-TIRADS 4 indicates intermediate suspicion of malignancy: (1) Solid hypoechoic composition without any of the three US features; (2) Partial cystic composition with any of three US features; (3) Solid iso/hyperechoic composition with any of three US features. K-TIRADS 3 indicates low suspicion of malignancy: (1) Partially cystic composition without any US feature; (2) Solid iso/hyperechoic composition without any US feature. K-TIRADS 2 indicates the nodule is benign: (1) Spongiform; (2) Partial cystic composition with comet-tail artifact; (3) Pure cyst. K-TIRADS 1 indicates no nodule.

### Key Points of Thyroid Nodular Assessment by the ACR TI-RADS (2017)

According to the ACR-TIRADS, the assessment of a thyroid nodule involves a comprehensive evaluation of its composition, echogenicity, shape (orientation) (8). In the scoring scheme, each sonographic feature is awarded from 0 to 3 points, and higher points indicate greater degrees of suspicious malignancy. The ACR-TIRADS categories were established according to the total score of a thyroid nodule, and there are five categories from benign to highly suspicious malignancy. A thyroid nodule with total score of 0, 2, 3, 4-6, and 7 and more is categorised as TIRADS 1, 2, 3, 4, and 5, respectively. Points are awarded for nodular composition (architecture) with these qualities: Cystic = 0, spongy = 0, mixed solid-cystic with dominant cystic = 1, solid = 2 and dominant solid or indeterminate = 2. Scores based on internal echogenicity are: Anechoic = 0, isoechoic = 1, hyperechoic = 1, indeterminate = 1, hypoechoic = 2, markedly hypoechoic = 3. Points awarded for shape (orientation) are: No point for wider than tall (A/T<1), and 3 points for taller than wide (A/T≥1). Points awarded for margin are: Smooth = 0, ill-defined = 0, irregular = 2, lobulated = 2, extrathyroidal extension = 3. Scores awarded for echogenic foci: none = 0, hyperechoic with comet-tail artifact = 0, macrocalcifications = 1, peripheral or rim hyperechoic = 2 and punctate hyperechoic = 3.

**Figure 1:**
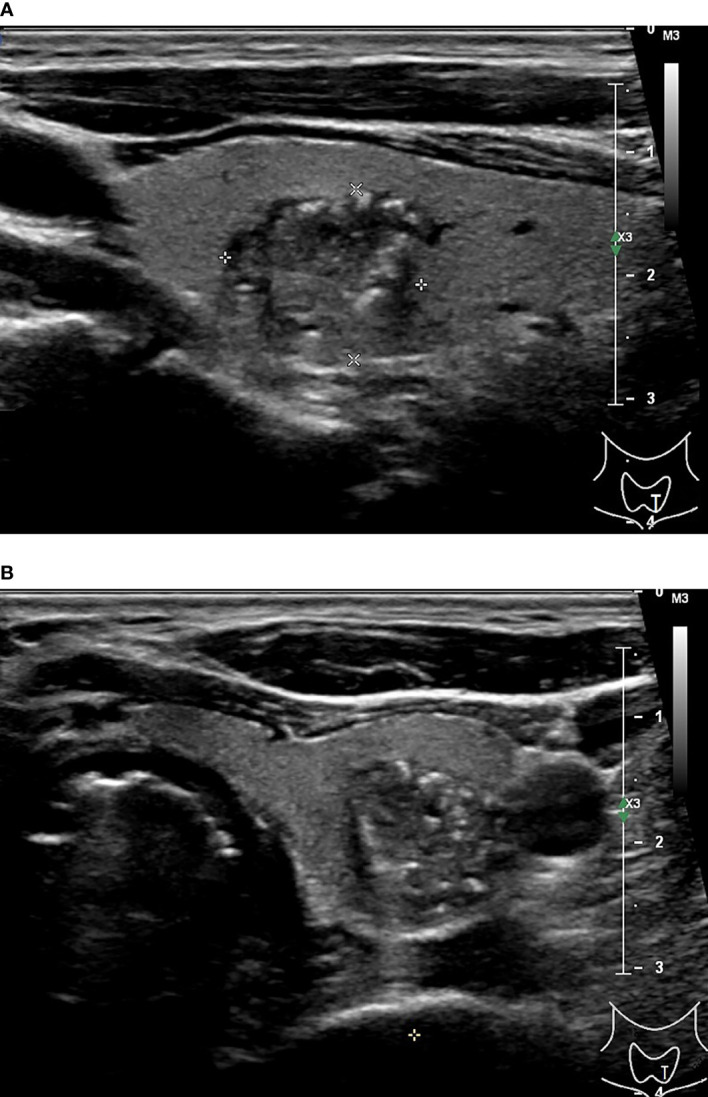
48-year-old man with thyroid papillary carcinoma. Sonographies of longitudinal scanning **(A)** and axial scanning **(B)** axial scanning show the nodule locates at the left lobe of the thyroid, characterized by 16.1 mm×13.8 mm×13.3mm in size, solid composition, irregular shape, A<T orientation in longitudinal view and A>T orientation in axial view, spiculated margin, heterogeneous iso/hypoechoic, with several punctate hyperechoic foci, and absence of posterior acoustic effect. C-TIRADS 5, K-TIRADS 5, ACR-TIRADS 5. Histopathology confirmed it is a papillary carcinoma.

**Figure 2:**
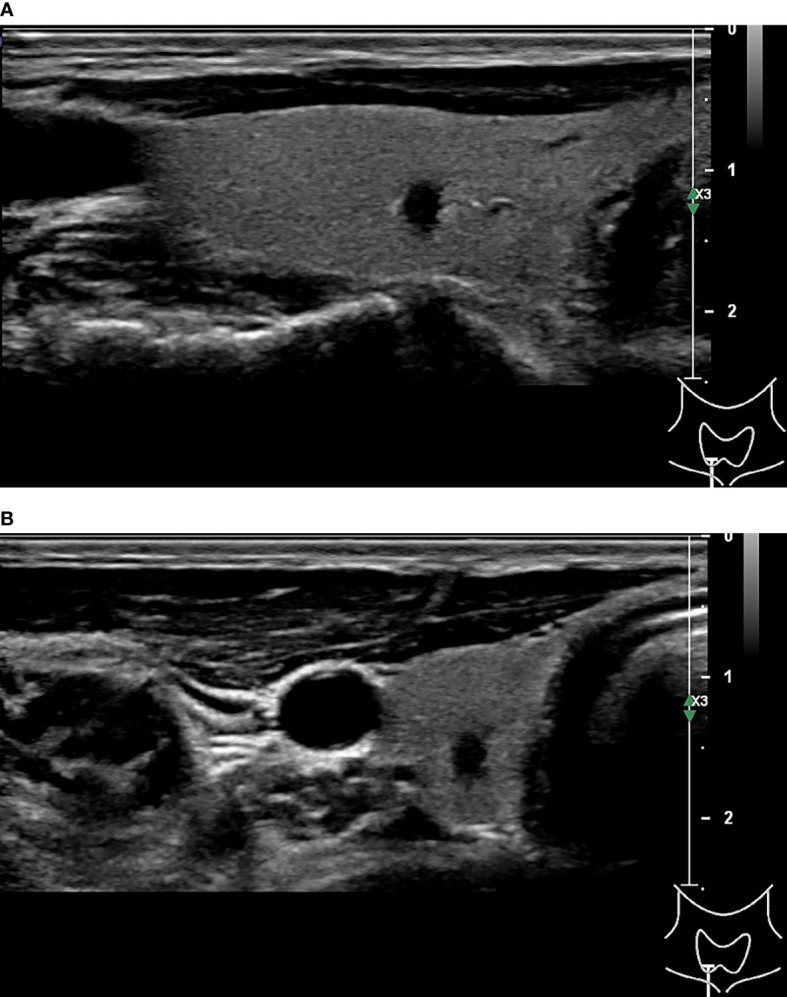
51-year-old man with thyroid papillary carcinoma. Sonographies of longitudinal scanning **(A)** and axial scanning **(B)** show the nodule locates at the right lobe of the thyroid, characterized by 4.9mm×3.3mm×3.5mm in size, solid composition, irregular shape, A>T orientation, markedly hypoechoic, well-defined margin, and absence of posterior acoustic effect. C-TIRADS 4C, K-TIRADS 5, ACR-TIRADS 5. Histopathology confirmed it is a papillary carcinoma.

**Figure 3:**
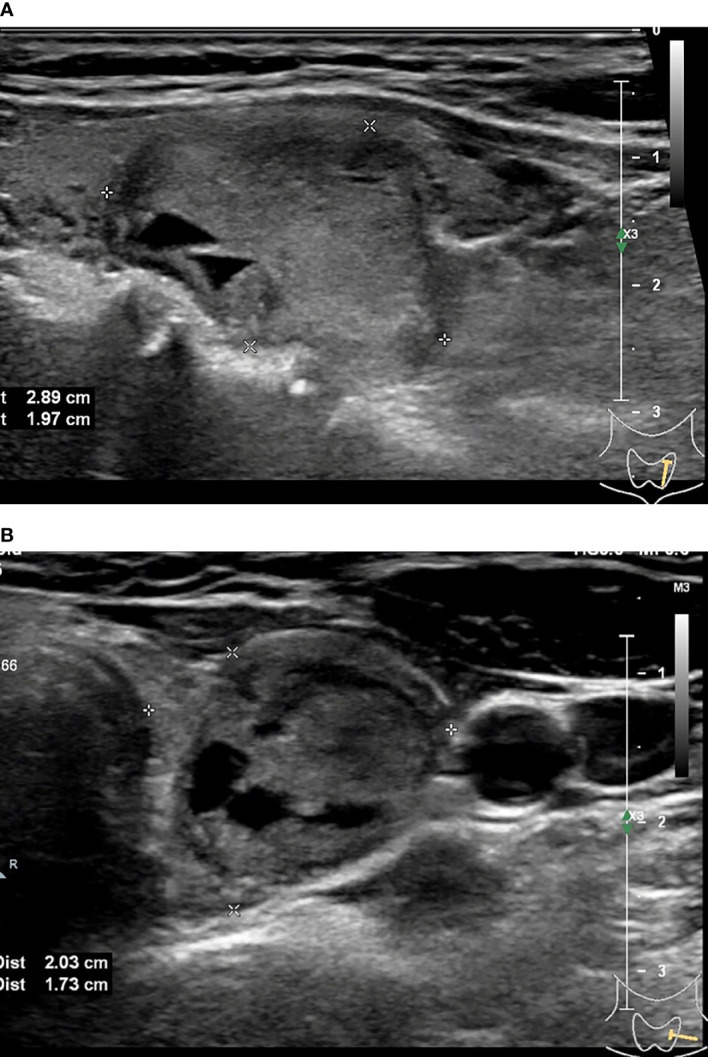
45-year-old woman with thyroid papillary carcinoma. Sonographies of longitudinal scanning **(A)** and axial scanning **(B)** show the nodule locates at the left lobe of the thyroid, characterized by 28.9mm×19.7mm×19mm in size, mixed solid-cystic composition, irregular shape, A<T orientation, hypoechoic and anechoic, discernible margin, and slight posterior acoustic enhancement. C-TIRADS 3, K-TIRADS 3, ACR-TIRADS 4. Histopathology confirmed it is a papillary carcinoma.

**Figure 4:**
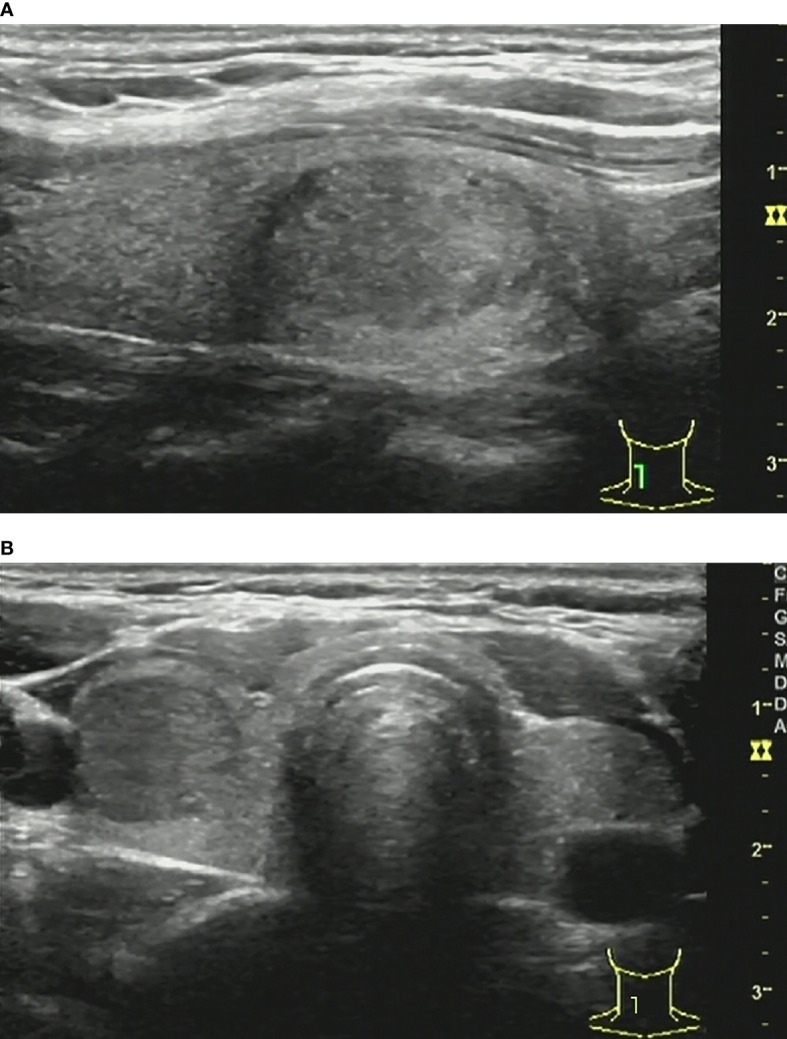
33-year-old woman with thyroid hyperplasia. Sonographies of longitudinal scanning **(A)** and axial scanning **(B)** show the nodule locates at the right lobe of the thyroid, characterized by 19.46mm×11.97mm×9.74mm in size, solid composition, elliptical shape, A<T orientation, almost isoechoic, well-defined margin, and absence of posterior acoustic effect. C-TIRADS 4A, K-TIRADS 3, ACR-TIRADS 3. Histopathology confirmed it is a thyroid hyperplasia.

**Figure 5:**
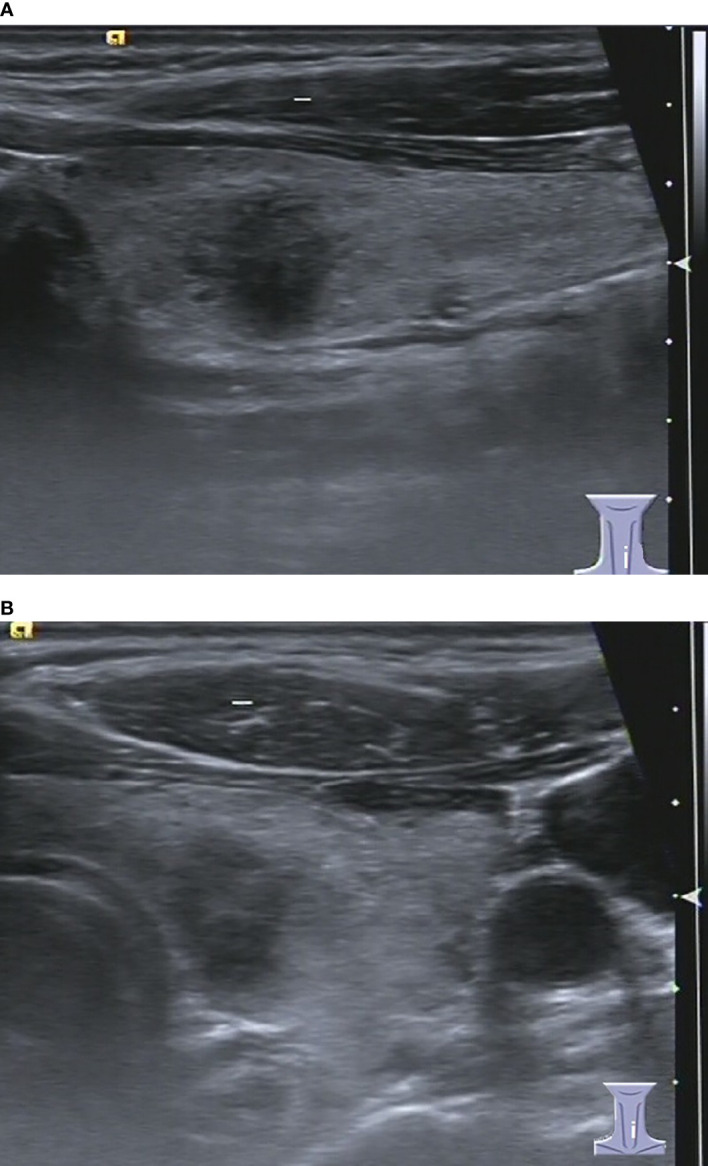
37-year-old woman with chronic lymphocytic thyroiditis. Sonographies of longitudinal scanning **(A)** and axial scanning **(B)** show the nodule locates at the left lobe of the thyroid, characterized by 9.8mm×9.2mm×9.4mm in size, solid composition, irregular shape, A>T orientation, hypoechoic, ill-defined margin, and absence of posterior acoustic effect. C-TIRADS 4C, K-TIRADS 5, ACR-TIRADS 5. Histopathology confirmed it is a chronic lymphocytic thyroiditis.

The thyroid nodule categorization was performed with reference to the C-TIRADS, K-TIRADS and ACR-TIRADS, the categorization was determined by two physicians with two and 16 years of experience in thyroid US through reviewing the US images in consensus, and they were blind to the histopathological results. Histopathological result was used as a reference to determine whether a thyroid nodule was benign or malignant. The histopathological study was made according to the criteria of the World Health Organization (17). US features of some thyroid nodules, scoring schemes and categorizations using three TIRADSs were illustrated on **Figures 1**–**5**.

#### Acquisition of Sonographies of the Thyroid Nodules

All patients with thyroid nodular lesions before surgery underwent thyroid US examination, using a linear array transducer with a frequency of 5-14 MHz and different US systems (Siemens Acuson S2000; Mindray DC 8 & 7; Aloka Prosound α-7 & α-10; Phillips EPIQ5 and GE Logiq E9). During the examination, the US systems were adjusted to small parts mode (thyroid gland); the patient took a supine position on a table without a pillow, with the neck fully exposed. The thyroid was scanned by different sections to detect any lesion. If a thyroid nodule was found, its location, shape, orientation(parallel or nonparallel), margin, border, size, architecture(composition) and internal echogenicity, posterior features, vascularity, and relation to abutting tissue were identified and scrutinized. Status of cervical lymph node was noted, with attention to the presence of punctate hyperechoic foci and calcification. The representative images were saved in Picture Archiving and Communications Systems (PACS).

### Statistical Analysis

Quantitative data with a normal distribution were expressed as mean and standard deviation (M ± SD), quantitative data that did not show a normal distribution and qualitative data were expressed as median (interquartile range, IQR) and percentile. The consistencies between C-TIRADS and K-TIRADS and ACR-TIRADS were studied, and the levels of malignancy risk stratification corresponding to each TIRADS were evaluated. The number of thyroid nodules and percentage of malignant nodules categorised as C-TIRADS 2, 3, 4A, 4B, 4C and 5, ACR-TIRADS 1, 2, 3, 4 and 5, and K-TIRADS 2, 3, 4 and 5 were calculated; and the correlation between the category and percentage of malignancy was determined, respectively. An independent samples T test was used to test quantitative data with a normal distribution. Qualitative data were analysed by a nonparametric test or a Chi-square test. The receiver operating characteristic (ROC) curve was drawn, the area under the (AUC) curve was obtained to evaluate the performances of the three TIRADSs for malignancy risk stratification, and the sensitivity, specificity, positive predictive value (PPV), and negative predictive value (NPV) were calculated. The highest value of accuracy (AUC) *of* ROC curve *of* each TIRADS was taken as the best threshold for malignancy risk stratification. Medcalc statistical software version 15.2.2 (Medcalc software BVBA, Ostend, Belgium) was used for statistical analyse, and *P* < 0.05 was considered a significant difference.

## Results

**Figure 6:**
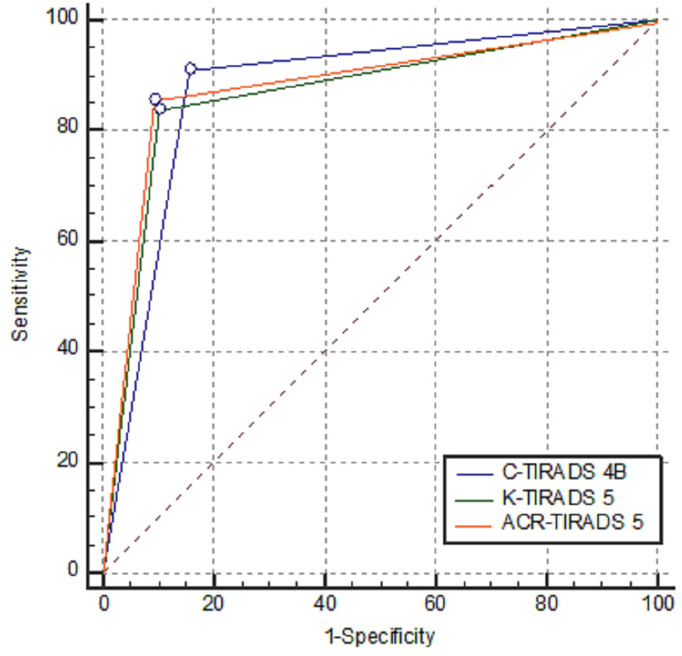
Comparisons of AUCs of C-TIRADS 4B, K-TIRADS 5 and ACR-TIRADS 5. There was no significant difference between each pair of them (*Z* value was 1.402, 0.306 and 1.004, respectively; and *P* value was 0.1610, 0.7593 and 0.3155, respectively).

Among the 1978 patients with 1982 thyroid nodules, there were 1306 benign nodules and 676 malignant nodules, and the demographic and ultrasound features of the patients with thyroid nodules are summarised in **Table 1**. Papillary carcinoma made up 97.48% of the total malignant nodules, and hyperplastic nodules made up 91.19% of the total benign nodules. Details of the distribution of pathologies of the thyroid nodules are listed in **Table 2**. The malignancy risk made up 1.09%, 2.14%, 10.34%, 49.28%, 88.19% and 85.29% of the total nodules that were categorised as C-TIRADS 2, 3, 4A, 4B, 4C and 5, respectively; 0.00%, 1.64%, 2.87%,18.71% and 82.22% of the total nodules that were categorised as ACR-TIRADS 1, 2, 3, 4 and 5, respectively;and 0.85%, 3.27%, 24.27% and 80.96% of the total nodules that were categorised as K-TIRADS 2, 3, 4 and 5, respectively. The correlation between the category of TIRADS and percentage of malignancy was 0.94 in the C-TIRADS, 1.00 in the ACR-TIRADS, and 1.00 in the K-TIRADS, respectively; as summarised in **Table 3**. The highest values of accuracy (AUC)*of* ROC curves *of* C-TIRADS 4B, K-TIRADS 5 and ACR-TIRADS 5 were taken as the optimised cut-off values (thresholds) for malignancy risk stratification, respectively. Using the optimised category of C-TIRADS 4B as the cut-off, 614 nodules were confirmed true positive (malignant lesions), 206 nodules were confirmed false positive (benign lesions), 1100 nodules were confirmed true negative (benign lesions), and 62 nodules were confirmed false negative (malignant lesions). Taking the optimised category of K-TIRADS 5 as the cut-off, 565 nodules were true positive (malignant lesions); 133 nodules were false positive (benign lesions); 1173 nodules were true negative (benign lesions); and 111 nodules were false negative (malignant lesions). Taking the optimised category of ACR-TIRADS 5 as the cut-off, 578 nodules were true positive (malignant lesions), 126 nodules were false positive (benign lesions), 1180 nodules were true negative (benign lesions), and 98 nodules were false negative (malignant lesions). The sensitivity, specificity, PPV, NPV and AUC by C-TIRADS 4B, K-TIRADS 5 and ACR-TIRADS 5 for malignancy risk stratification of thyroid nodules were 90.83%, 84.23%, 74.88%, 94.66% and 0.88; 83.58%, 89.82%, 80.95%, 91.36% and 0.87; and 85.50%, 90.35%, 82.10%, 92.33% and 0.88, respectively. There was no significant difference between them when comparing AUCs between any two of them (*P*>0.05 for all). These are summarised in **Table 4** and **Figure 6**. There was significant difference in the comparison between any two of the C-TIRADS 4C, 4B, and 4A for AUC (all *P*<0.001).

**Table 1:** Demographic and ultrasound features of the patients with thyroid nodules.

Characteristics	Benign nodules (n=1306)	Malignant nodules (n=676)	*P value*
Age (year)			<0.0001
Mean	46.41 ± 12.65	43.93 ± 11.93	
Range	7-75	7-82	
Sex			0.5211
Male (n,%)	291 (22.28)	160 (23.67)	
Female (n,%)	1015 (77.72)	516 (76.33)	
Size (mm)			<0.0001
Mean	21.34 ± 11.66	11.71 ± 8.47	
Range	(2-79)	(2-73)	
Distribution of sizes			<0.0001
<10mm (n,%)	131 (10.03)	287 (42.46)	
≥10mm (n,%)	1175 (89.97)	389 (57.54)	
Number			
Single (n,%)	312 (23.89)	162 (23.96)	0.9852
Multiple (n,%)	994 (76.11)	514 (76.04)	
Composition			<0.0001
Cystic/spongiform (n,%)	39 (2.99)	0 (0.00)	
Mixed cystic and solid (n,%)	635 (48.62)	23 (3.40)	
Solid (n,%)	632 (48.39)	653 (96.60)	
Echogenicity			<0.0001
Anechoic (n,%)	32 (2.45)	0 (0.00)	
Iso/hyperechoic (n,%)	604 (46.25)	56 (8.28%)	
Hypoechoic (n,%)	648 (49.62)	526 (77.81%)	
Markedly hypoechoic (n,%)	22 (1.68)	94 (13.91%)	
Shape			<0.0001
Wider-than-tall (n,%)	1262 (96.63)	355 (52.51)	
Taller-than-wide (n,%)	44 (3.37)	321 (47.49)	
Margin			<0.0001
Smooth/ill-defined (n,%)	1246 (95.41)	319 (47.19)	
Lobulated/irregular (n,%)	58 (4.44)	308 (45.56)	
Extrathyroid extension (n,%)	2 (0.15)	49 (7.25)	
Echogenic foci			<0.0001
None or large comet-tail artifacts (n,%)	1055 (80.78)	160 (23.66)	
Macrocalcifications (n,%)	90 (6.89)	25 (3.70)	
Peripheral calcifications (n,%)	22 (1.69)	7 (1.04)	
Punctate echogenic foci (n,%)	139 (10.64)	484 (71.60)	

**Table 2:** Distribution of pathologies of the thyroid nodules.

Pathology		Number (%)
Benign nodule	Hyperplastic nodules	1191 (91.19)
	Follicular adenoma	49 (3.75)
	Hürthle-cell adenoma	14 (1.07)
	Chronic lymphocytic thyroiditis	41 (3.14)
	Toxic nodular goiter	3 (0.23)
	Granulomatous thyroiditis	8 (0.62)
	Total	1306 (100.00)
Malignant nodule	Papillary carcinoma	659 (97.48)
	Medullary carcinoma	9 (1.33)
	Follicular carcinoma	6 (0.89)
	Anaplastic carcinoma	2 (0.30)
	Total	676 (100.00)

**Table 3:** Distributions of benign and malignant nodules in different categories of TIRADSs and correlations.

TIRADS	Benigndistribution (n)	Malignantdistribution (n)	Sum and percentile (n,%)	Malignancy risk (%)	Coefficient of malignancy risk and TIRADS
C-TIRADS					0.943
2	90	1	91 (4.59)	1.09	
3	594	13	607 (30.61)	2.14	
4A	416	48	464 (23.41)	10.34	
4B	141	137	278 (14.03)	49.28	
4C	60	448	508 (25.63)	88.19	
5	5	29	34 (1.72)	85.29	
Total	1306	676	1982 (100)		
ACR-TIRADS					1.00
1	34	0	34 (1.72)	0.00	
2	300	5	305 (15.39)	1.64	
3	508	15	523 (26.39)	2.87	
4	339	78	417 (21.04)	18.71	
5	125	578	703 (35.47)	82.22	
Total	1306	676	1982 (100)		
K-TIRADS					1.00
2	116	1	117 (5.90)	0.85	
3	798	27	825 (41.67)	3.27	
4	259	83	342 (17.26)	24.27	
5	133	565	698 (35.22)	80.96	
Total	1306	676	1982 (100)		

**Table 4:** Diagnostic performances of three TI-RADSs by different cut-off of category.

Cut-off of category	Sensitivity (%,n)	Specificity (%,n)	PPV (%,n)	NPV (%,n)	AUC
C-TIRADS 4A	97.93 (662/676) [96.55-98.86]	52.37 (684/1306) [49.62-55.11]	51.56 (662/1284) [48.78-54.32]	97.99 (684/698) [96.66-98.90]	0.75 [0.73-0.77]
C-TIRADS 4B C-TIRADS 4C C-TIRADS 5 K-TIRADS 4	90.83 (614/676)	84.23 (1100/1306)	74.88 (614/820)	94.66 (1100/1162)	0.88
	[88.40-92.90]	[82.13-86.16]	[71.76-77.81]	[93.21-95.89]	[0.86-0.89]
	70.86 (479/676)	95.02 (1241/1306)	88.05 (479/544)	86.30 (1241/1438)	0.83
	[67.27-74.26]	[93.71-96.14]	[85.03-90.66]	[84.41-88.04]	[0.81-0.84]
	4.73 (32/676)	99.62 (1301/1306)	86.49 (32/37)	66.89 (1301/1945)	0.52
	[3.26-6.62] 95.86 (648/676) [94.07-97.23]	[99.11-99.88] 69.98 (914/1306) [67.42-72.46]	[71.23-95.46] 62.31 (648/1040) [59.28-65.26]	[64.75-68.98] 97.03 (914/942) [95.73-98.02]	[0.50-0.54] 0.83 [0.81-0.85]
K-TIRADS 5	83.58 (56/676) [80.57-86.29]	89.82 (1173/1306) [88.05-91.40]	80.95 (565/698) [77.83-83.79]	91.36 (1173/1284) [89.68-92.84]	0.87 [0.85-0.88]
ACR-TIRADS 4	97.04 (656/676)	64.62 (844/1306)	58.68 (656/1118)	97.69 (844/864)	0.81
	[95.47-98.18]	[61.96-67.22]	[55.73-61.58]	[96.45-98.58]	[0.79-0.82]
ACR-TIRADS 5	85.50 (578/676) [82.62-88.07]	90.35 (1180/1306) [88.62-91.90]	82.10 (578/704) [79.07-84.87]	92.33 (1180/1287) [90.73-93.73]	0.88 [0.86-0.89]

C-TIRADS, The Superficial Organ and Vascular Ultrasound Group of the Society of Ultrasound in Medicine of Chinese Medical Association version of thyroid imaging, reporting and data system; K-TIRADS, The Korean Thyroid Imaging Reporting and Data System by The Korean Society of Thyroid Radiology; ACR-TIRADS, American College of Radiology version of thyroid imaging, reporting and data system; PPV, Positive predictive value; NPV, Negative predictive value; AUC, Area under the ROC curve; Variables in parentheses are numbers; Variables in brackets are 95% confidential intervals.

## Discussion

Our study showed that the C-TIRADS has high clinical significance for stratifying the malignancy risk of thyroid nodules, and the AUC with a cut-off of C-TIRADS 4B is 0.88. Which is a little higher than that of K-TIRADS 5, equal to that of ACR-TIRADS 5, and higher than that 0.83 of C-TIRADS 4C, 0.52 of C-TIRADS 5, and 0.75 of C-TIRADS 4A. With C-TIRADS 4B as the stratification threshold, the sensitivity and specificity in the stratification of malignant thyroid nodules were 90.83% and 84.23%, respectively, which are similar to the results from K-TIRADS 5 and ACR-TIRADS 5. AUC of C-TIRADS 5 in our study is much lower than the AUCs of C-TIRADS 5, K-TIRADS 5 and ACR-TIRADS 5 in the study by Zhou et al. (18), and the AUC of C-TIRADS 4B in our study is a little higher than the AUCs of K-TIRADS 5 and ACR-TIRADS 5 in the study by Zhou et al. (18). The AUC of ACR-TIRADS 5 in this study is 0.88, which is higher than those in the previous studies of accuracy of 0.52 and AUC of 0.835 and 0.864 (19–21).

In our study, the NPV was 94.66% when using the C-RADS 4B as the optimal cut-off, which was higher than that of the K-TIRADS (91.36%) and ACR-TIRADS 5 (92.23%) at their optimal cut-off; and was a little lower than the result of 96.4% reported by Zhou et al. (18). It suggests that using C-RADS 4B as cut-off may spare more unuseful fine needle aspiration(FNA) or coarse needle biopsy.

The C-TIRADS has several advantages: First, it is aimed at stratifying the malignancy risk, and clinical management is determined by a clinician with reference to other information. The malignancy risk in C-TIRADS 5 is over 90%, which are substantial higher than those of K-TIRADS (over 60%) and ACR-TIRADS(over 20%). The orientation of ACR-TIRADS is used to stratify the malignancy risk and FNA and/or follow-up, and management should wait for the results of FNA. Second, the US features for stratifying the malignancy risk adopted for the C-TIRADS may be more scientific, since these features have been optimised and evaluated by building a multivariate logistic regression model with forward stepwise selection and significant US features were included in the final logistic regression analysis (18). This was done instead of scoring every US feature based expert opinion. Third, the three TIRADSs have almost the same interpretations of US features, but the schemes are different. The scoring scheme of C-TIRADS is simpler and clearer (8, 9). For example, fine punctate microcalcification is given a value of 1 in the C-TIRADS, awarded 3 points in the ACR-TIRADS, and set as an important indicator of highly suggestive malignancy in the K-TIRADS. The hyperechoic foci with comet-tail artifact are counted as -1 in C-TIRADS, awarded 0 point in ACR-TIRADS, and regarded as an indicator suggestive of benign nodule. The scoring scheme of ACR-TIRADS involves 23 US features with different weighting of scores that suggest malignancy; the scheme of K-TIRADS involves three marked US features highly suspicious of malignancy and twenty or so other US features, without awarding a score. The scoring scheme of C-TIRADS involves counting six US features that are highly suggestive of malignancy or benignity. Other features are precluded. Fourth, the C-TIRADS can spare radiologist time for cumulating scores, and it has more clinical practicability.

The C-TIRADS also has some weaknesses: First, all thyroid nodules with solid composition are counted as 1 in the C-TIRADS, which will lead to many benign nodules with scores of 1 or more, be categorised as C-TIRADS 4A or 4B, and increase the false negative effect. Second, a very (markedly) hypoechoic feature has 1 counting value suggestive of malignancy in the C-TIRADS. However, many malignant nodules do not present markedly hypoechoic feature. For example, Zhou et al. reported it was 27.79% (157/565) (9), but it made up of only 13.9% (94/676) in our study. This offsets the total score of a nodule and leads to an increase in the rate of false negatives. Third, the hypoechoic feature that occurs more frequently in malignant nodules has not been recruited for counting and categorising in the C-TIRADS. It made up of 59.82% (338/565) in the study by Zhou et al. (9) and 77.81% (526/676) in our study. This offsets the total score of a nodule and leads to an increase in the rate of false negatives. Fourth, the C-TIRADS has six categories, four of which are based on the data provided by the Chinese Artificial Intelligence Alliance for Thyroid and Breast Ultrasound, and the other two are otherwise, which may affect the soundness and robustness of the C-TIRADS (9).

According to the C-TIRADS guidelines, the malignancy risk of C-TIRADS 5, 4C, 4B, 4A, 3, 2, 1 were over 90%, over 50% and equal or fewer than 90%, over 10% and equal or fewer than 50%, over 2% and equal or fewer than 10%, fewer than 2%, 0% and 0%, with high consistency and limited deviation (9). Our results for C-TIRADS category 5, 4C, 4B, 4A, 3 and 2 (absence of thyroid nodules for category 1 and 6) were 85.29%, 88.19%, 49.28%, 10.34%, 2.14% and1.09%; the correlation between the category and malignancy risk was 0.94; and AUCs of C-TIRADS 5, 4C, 4B and 4A were 0.52, 0.83, 0.88 and 0.75, respectively. These indicate the correlation was excellent, but the AUC was inconsistency and great deviation compared to the Chinese guidelines, especially the C-TIRADS 5. The reasons may be that many malignant nodules do not present some US features that are suggestive of malignant nodules, while substantial weight is placed just on these features, including extra-thyroid extension margin, marked hypoechogenicity and taller-than-wide shape, and these lead to a lower total score, so the nodules cannot be categorised as C-TIRADS 5.

According to the Korea’s recommendations, the malignancy risk of K-TIRADS 5, 4, 3, 2, 1 were over 60%, over 15% and equal or less than 60%, over 3% and equal or less than 15%, less than 3%, and 0 (7). Our results of K-TIRADS 5, 4, 3 and 2 (absence of thyroid nodules for 1 category) were 80.96%, 24.27%, 3.27%, and 0.85%; the correlation between the category and malignancy risk was 1.00; and AUCs of K-TIRADS 5 and 4 were 0.87 and 0.83, respectively. The correlation was perfect, but the AUC was not consistent with the recommendations. This means that many thyroid nodules with K-TIRADS 4 and 5 have a higher malignancy risk than the ranges stated in the recommendations, and the K-TIRADS has higher predictive ability than its original data.

The main aim of the ACR-TIRADS category was to determine recommendations for FNA and follow-up, with a low threshold of malignancy risk estimated at 2% or less for ACR-TIRADS 1 to greater than 20% for ACR-TIRADS 5. In our study, the malignancy risk of ACR-TIRADS 5, 4, 3, 2, 1 category was 82.22%, 18.71%, 2.87%, 1.64% and 0.00%; the correlation between the category and malignancy risk was 1.00; and the AUC of 0.88 was high. The correlation was perfect, but the AUC was not consistent with the recommendations. This means that many thyroid nodules with ACR-TIRADS 5 have much higher than 20% malignancy risk, as reported by other studies (18–22).

Previous study has shown that there was wide variability in the description of some US features, while interobserver agreement among different TIRADSs was substantial to almost perfect (23). In our study, the best thresholds for assessing malignant thyroid nodules by the C-TIRADS, K-TIRADS and ACR-TIRADS were C-TIRADS 4B, K-TIRADS 5 and ACR-TIRADS 5, respectively. The reason that C-TIRADS 4B performed better than C-TIRADS 4C and 5 may be that some malignant nodules do not present sufficient US features to indicate malignancy and some benign nodules present more US features, suggesting malignancy. This leads to inappropriate scoring and poorer diagnostic performance. Similar phenomena have occurred in other studies. For example, Basha et al. found that in their multi-centre prospective study on the validity of ACR-TIRADS based 948 thyroid nodules, the best cut-off value for predicting malignant thyroid nodules was > ACR-TIRADS 3, other than ACR-TIRADS 5 (24).

In our study, the C-TIRADS 4B, K-TI-RADS 5 and ACR-TIRADS 5 showed good sensitivity and specificity in risk stratification of thyroid nodules. This means that they are better at differentiating malignant thyroid nodules from benign ones. This suggests that the three TIRADSs for thyroid nodules are beneficial for patients and radiologists. Because the thyroid cancer is an indolent tumor, the progress is usully slow, so for a lower malignancy risk thyroid nodule, it’s not so urgent to perform FNA or a biopsy, and follow-up may be an alternative. These were supported by Grani et al. that patients with presumably benign thyroid nodules assessed by TIRADS can be safely followed with less intensive protocols (25). The C-TIRADS 5 presented excellent specificity, at 99.62%. This means that if a thyroid nodule presents C-TIRADS 5, there is little probability that it is benign. However, a sensitivity of 4.73% handicaps its power in malignancy risk stratification. The ACR-TIRADS and K-TI-RADS have been validated by many studies, but the C-TIRADS 2020 version is a recently launched system (18–27).

Prospectively, because the three investigated TIRADSs had not considered the locations of the thyroid nodules, cervical lymph nodes, epidemic factors, contrast-enhanced US features, US elastography, artificial intelligence, and so on. Some experts have suggested adding one or more of these variables to extend and augment these systems and to improve their risk stratification efficiency (14, 28–32). Wang, et al. reported that contrast-enhanced US combining with conventional US in differentiating ACR TI-RADS category 4 and 5 nodules with non-hypovascular may improve the malignancy risk stratification of non-hypovascular thyroid nodules (28). Celletti et al. found that adding strain elastosonography of Strain Ratio to K-TIRADS assessment can significantly increase its sensitivity and negative predictive value (30). A study by Wildman-Tobriner et al. showed that an artificial intelligence-optimized ACR-TIRADS can slightly improve its specificity and maintain sensitivity. Additionally, it simplifies US feature assignments, which may improve ease of use (32).

This study has some limitations: (1) The surgery and histopathological results are used as the gold standard, instead of combining surgery and FNA cytology, which cannot fully include benign lesions, and may inevitably induce selection bias. (2) The absence of assessment of interobserver agreement for the US images acquisition of the thyroid nodules by different operators and different US systems, which may affect the homogeneicity of the images. (3) In this study, the malignant nodules are mainly papillary carcinoma (97.48%), while the benign nodules are hyperplastic nodules (91.19%), and the pathological types are relatively narrow. Therefore, in the future, more study should be done to evaluate the risk stratification efficiency for medullary carcinoma, follicular carcinoma and anaplastic carcinoma.

The strengths of this study are that (1) It is a relative new study for the validation of the latest released C-TIRADS; (2) The sample is large; (3) The pathological natures of the thyroid nodules had been confirmed by histopathology, thyroid nodules with FNA cytology only were excluded, so the final diagnosis of the thyroid nodules was reliable and sound; (4) The validation of C-TIRADS was compared with the widely validated ACR-TIRADS and K-TIRADS, and the results and conclusion are sufficiently informative. The weaknesses of this study are that (1) The sample was rendered from a single medical centre, and (2) The study design was retrospective, which may have potential of causing bias for the study.

In summary, the C-TIRADS has outstanding performance in the malignancy risk stratification of thyroid nodules by the optimised cut-off value, which is comparable to that in K-TIRADS and ACR-TIRADS.

## Data Availability Statement

The original contributions presented in the study are included in the article/supplementary material. Further inquiries can be directed to the corresponding author.

## Ethics Statement

The studies involving human participants were reviewed and approved by The Ethics Committee of The First Affiliated Hospital of Hainan Medical University. Informed consent was obtained from all patients for the use of US images and related information for the purpose of teaching and scientific research during the examination. 

## Author Contributions

(1) Contributor SW: Concepts, design, data acquisition and analysis, statistical analysis, manuscript preparation, and guarantor. (2) Contributor QC: Data acquisition and analysis, and statistical analysis. (3) Contributor ML: Data acquisition and analysis, and manuscript preparation. All authors: Final approval of the version to be published; and agreement to be accountable for all aspects of the work in ensuring that questions related to the accuracy or integrity of any part of the work are appropriately investigated and resolved.

## Funding 

This study was financially supported by grant from the National Natural Science Foundation of China (grant no. 81560290).

## Conflict of Interest

The authors declare that the research was conducted in the absence of any commercial or financial relationships that could be construed as a potential conflict of interest.

## Publisher’s Note

All claims expressed in this article are solely those of the authors and do not necessarily represent those of their affiliated organizations, or those of the publisher, the editors and the reviewers. Any product that may be evaluated in this article, or claim that may be made by its manufacturer, is not guaranteed or endorsed by the publisher.
